# Sand fly (Diptera, Psychodidae, Phlebotominae) abundance and diversity in areas affected by the São Francisco River transposition project in Ceará State, Brazil

**DOI:** 10.1186/s13071-017-2333-z

**Published:** 2017-08-29

**Authors:** Júlia dos Santos Silva, Lindemberg Caranha, Fabrício Kássio Moura Santos, Antonio Pereira dos Santos, Luiz Osvaldo Rodrigues da Silva, Elizabeth Ferreira Rangel

**Affiliations:** 1 Laboratório Interdisciplinar de Vigilância Entomológica em Diptera e Hemiptera, Instituto Oswaldo Cruz, FIOCRUZ, Av. Brasil, 4365, 21040-360, Manguinhos, Rio de Janeiro, Brazil; 2 Secretaria de Saúde do Estado do Ceará, Fortaleza, Brazil

## Abstract

**Background:**

Entomological surveillance of sand fly vectors was carried out to support leishmaniasis prevention and control measures in areas affected by the São Francisco River Transposition Project.

**Methods:**

Sand flies were collected monthly between May 2011 and December 2014 in seven municipalities: Missão Velha, Brejo Santo, Lavras da Mangabeira, Iguatu, Mauriti, Jaguaribe and Jaguaretama, in dwellings, peridomicile and forest areas for three consecutive days, for a period of 12 h each day (18:00 to 06:00 h). Differences in species composition between sites were tested with Shannon’s diversity index, the similarity between habitats was estimated by the Sørensen’s qualitative similarity index and, for the most abundant species in each municipality, a standardized index of species abundance was applied. The influence of climatic factors on sand fly population densities was analyzed using Spearman’s correlation coefficients.

**Results:**

A total of 214,213 sand fly specimens belonging to 18 species were captured. The most abundant species in all municipalities was *Lutzomyia longipalpis* (Lutz & Neiva, 1912). The municipalities of Mauriti and Missão Velha stand out in terms of high species richness, with the latter exhibiting the greatest diversity. The number of sand flies in the Iguatu, Jaguaribe and Jaguaretama municipality was higher during the rainy months, whereas the populations declined in the drier months; the sand fly population density in other municipalities (Missão Velha, Brejo Santo, Lavras de Mangabeira and Mauriti) showed negative correlation with rainfall.

**Conclusions:**

This study confirms the presence of several *Leishmania* spp. vectors in the seven municipalities affected by the São Francisco River Transposition Project, with *Lu. longipalpis* being the most abundant species at all study sites. Vector populations in these municipalities should be monitored, ultimately to assess the associations between environmental changes and sand fly population dynamics and leishmaniasis transmission risk.

## Background

Leishmaniasis poses significant public health problems in Brazil. Both American cutaneous leishmaniasis (ACL) and American visceral leishmaniasis (AVL) are expanding and new epidemiological scenarios are emerging. AVL, in particular, has become urbanized, becoming endemic in medium and large cities in different regions of the country [1, 2]. Approximately 90% of human AVL cases registered in South America occur in Brazil [2, 3]. ACL has been recorded in every state in Brazil, and is currently in a territorial expansion phase and changing its epidemiological profile [1]. Studies suggest that the geographic expansion of leishmaniasis stems from anthropic activities, by altering the environmental conditions that favor sand fly populations and increase their contact with humans [3, 4].

In northeastern Brazil, AVL is endemic to the State of Ceará (CE), with outbreaks recorded in several municipalities. Fortaleza, Sobral, Juazeiro, Barbalha and Caucaia are priority municipalities for control activities; in fact, according to the classification by the Ministry of Health, Fortaleza is an intense transmission city [5, 6]. ACL, caused by *Leishmania* (*Viannia*) *braziliensis* (Vianna, 1911), is unquestionably one of the major public health concerns in Ceará. It occurs in several areas, especially in mountainous regions and those adjacent to the coast, with municipalities such as Uruburetama, Itapajé, Barbalha and Missão Velha being the areas at greatest risk [7].

The São Francisco River Transposition Project aims at transforming the socio-economic reality in northeast Brazil by ensuring water supply to several states, including Ceará, where the transposition began in 2008. However, some negative impacts have been ascribed to this project, such as modifications of ecosystems that favor the expansion of vector populations that consequently lead to increase in vector-borne diseases such as leishmaniasis.

Both AVL and ACL are endemic to the State of Ceará; thus, the Ministry of Health recommended an evaluation of the populations of sand fly vectors, and monitoring the emergence of new likely sites of infection in municipalities under directly influence in the area affected by this (still ongoing) work. Entomological surveillance aids in increasing the knowledge of the sand fly fauna in general, and indicates the occurrence of potential leishmaniasis vectors.

The present study focused on entomological surveillance of sand flies aiming to support leishmaniasis prevention and control measures in the areas affected by the project. This enabled a global analysis of sand fly communities in dwellings, peridomicile and forest areas, and allowed characterization of the fauna in seven municipalities of Ceará under direct influence of the São Francisco River Transposition Project, in terms of species richness, diversity, dominance, abundance and similarity.

## Methods

**Fig. 1:**
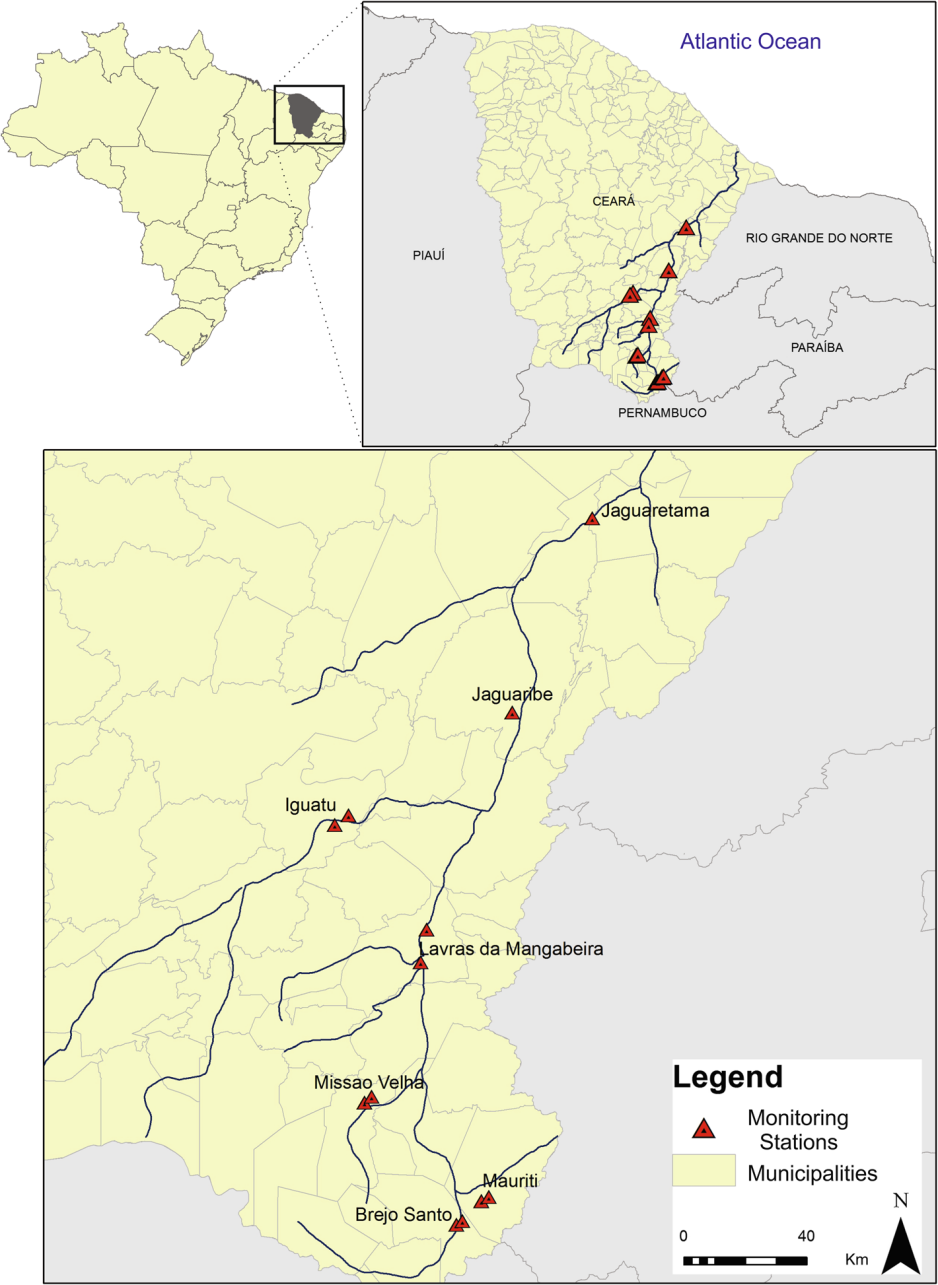
Location of São Francisco River Transposition Project area in Brazil, highlighting each of the municipalities studied

Monitoring of sand flies was performed in the areas of influence of Section I, in the North axis of the São Francisco River Transposition Project (Fig. 1).

The current physical execution rate of the São Francisco River Transposition Project is 89.9% (90.7 and 88.7% in the North and East axes, respectively), and three of the project’s pumping stations have engines in operation. The impact area of the present study is included in Key Goal 3 N (81 km) of the North axis, extending from Boi II reservoir in the municipality of Brejo Santo, Ceará State to the Engenheiros Ávidos reservoir in the municipality of Cajazeiras, Paraíba State, with 94% of physical implementation [8].

**Fig. 2:**
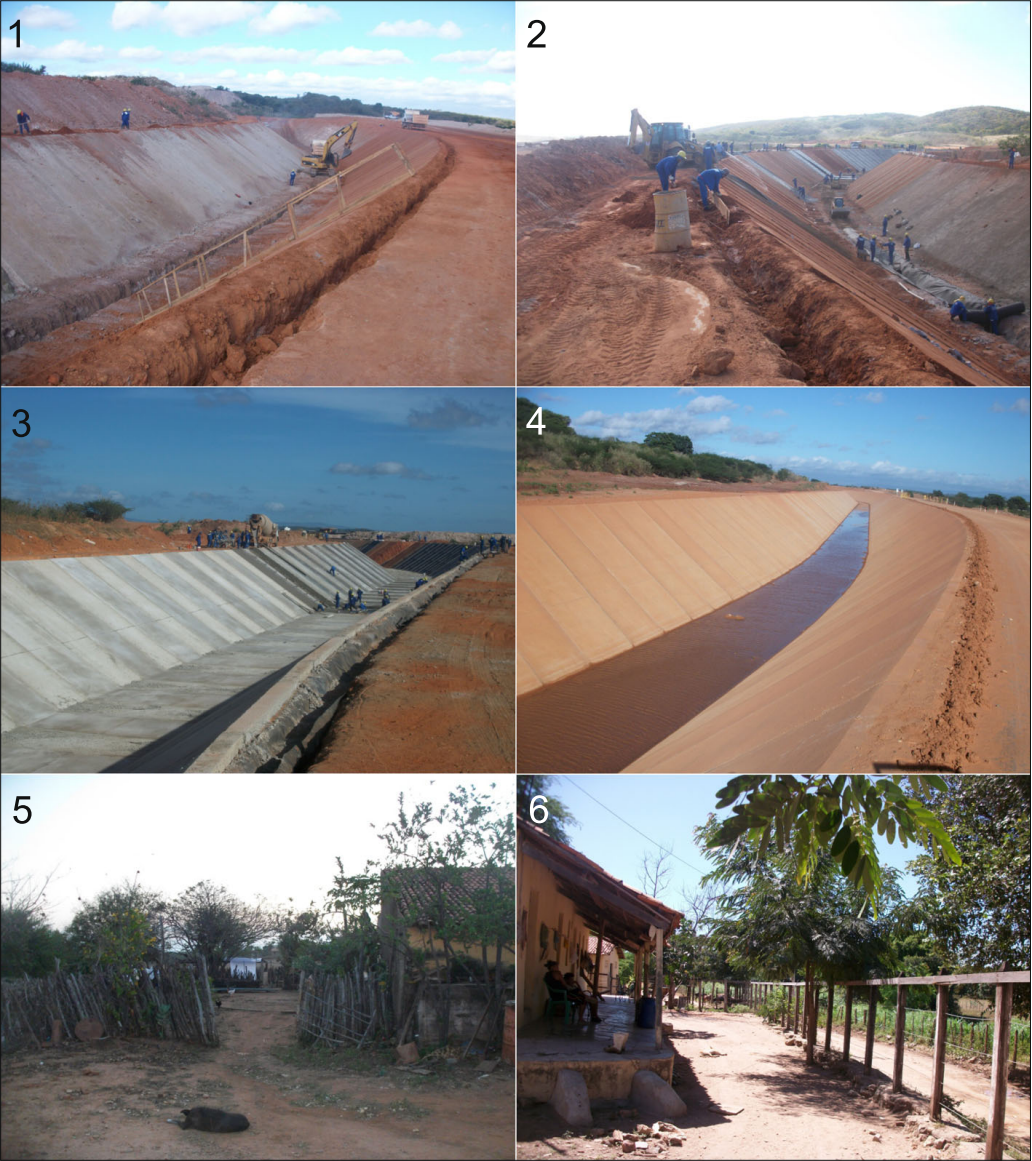
Illustrations of some of the monitoring stations in the São Francisco River Transposition Project area in Brazil, Ceará, Brazil: 1–3, impacted area in Mauriti municipality: period of the construction; 4, impacted area in Mauriti municipality: part of the construction concluded; 5, typical peridomicile in Iguatu municipality; 6, typical peridomicile in Missão Velha municipality

Twelve monitoring stations (MSs) were established in seven municipalities: Missão Velha (07°12′45.6″S, 39°06′57.5″W and 7°11′42.8″S, 39°05′42.3″W), Brejo Santo (7°34′13.6″S, 38°50′47.7″W and 7°33′35.3″S, 38°49′41.9″W), Lavras da Mangabeira (6°42′17.2″S, 38°55′58.8″W and 6°48′02.9″S, 38°57′04.9″W), Iguatu (6°22′10.3″S, 39°09′45.6″W and 6°23′52.1″S, 39°12′10.6″W), Mauriti (07°30′04.1″S, 38°46′24.2″W and 07°29′22.5″S, 38°45′05.7″W), Jaguaribe (6°04′06.4″S, 38°40′87.9″W), and Jaguaretama (6°41′46.7″S, 38°56′50.7″W). The MSs were established within the prioritization logic of the Department of Health of the State of Ceará on leishmaniasis surveillance and control actions (Figs. 1 and 2).

Samples were obtained monthly from May 2011 to December 2014, in dwellings, peridomicile, and forest areas. The forests were located about 500 m distant from the peridomicile, with the vegetation in each area being classified as follows: Missão Velha and Brejo Santo, deciduous thorny forest (*Floresta caducifólia espinhosa*); Mauriti, rainforest subtropical deciduous forest (*Floresta subcaducifólia tropical pluvial*); Lavras de Mangabeira and Iguatu, dense shrub caatinga (*Caatinga arbustiva densa*); Jaguaribe and Jaguaretama, open shrub caatinga (*Caatinga arbustiva aberta*). Three HP-model CDC light traps [9] were employed in each MS, at a height of approximately 1 m from the ground, for three consecutive days, for a period of 12 h every day (18:00 to 06:00 h). The taxonomic diagnosis of sand flies followed Galati’s proposal [10, 11]. Abbreviations of generic and subgeneric names follow those proposed by Marcondes [12].

The Shannon’s diversity index (H′) was used in the evaluation and the comparison of differences in composition within the sand fly community in each area of the study [13]. To assess the presence of significant differences between the diversity indices, Student’s *t*-test was applied, with significance level of 5%, using the software DivEs® [14]. The diversity index (H′) was chosen for its suitability in studies comprising random samples of species within a community or subcommunity. Species dominance (D%) was defined by: D% = (i/t) × 100, where i is the total of individuals of a particular species and t is the total number of specimens collected. To evaluate the most abundant species in each MS, we applied the index of species abundance (ISA), which was normalized to values between zero and one by the standardized index of species abundance (SISA), according to the definitions proposed by Roberts & Hsi [15]. SISA values close to 1 correspond to the most abundant species. These indices were calculated using the formulae:

$$ \mathrm{ISA}=\mathrm{a}+\mathrm{Rj}/\mathrm{K};\mathrm{and}\ \mathrm{SISA}=\mathrm{c}\hbox{-} \mathrm{ISA}/\mathrm{c}\hbox{--} 1, $$

where a is the number of samples in which the species was absent multiplied by c; c is the highest value of *n* obtained, considering all samples, plus 1; for each sample, species should be ranked from 1 to n (1 assigned to the most frequent species); Rj is the sum of the positions of each species; and K is the number of samples.

The similarity between habitats with respect to the number of species was estimated by the Sorensen-Dice qualitative similarity index (SI), based on the presence or absence of the species [16]. Kruskal-Wallis analysis, with significance level of 5%, was used to evaluate the occurrence of significant difference between sand fly populations in dwellings, peridomicile and forest areas, for each municipality, using the software IBM® SPSS® Statistics Version 23.

The monthly frequency was represented as total number of specimens captured per municipality. These data were obtained from the most abundant species, as well as the main vectors of leishmaniasis, considering the total numbers of specimens in dwellings, peridomicile and forest areas of each municipality, per month during the 44 months of captures.

The influence of rainfall on sand fly population densities was analyzed by Spearman’s correlation coefficient with 95% and 99% confidence level, using the software IBM® SPSS® Statistics Version 23. The rainfall data were provided by FUNCEME (Fundação Cearense de Meteorologia e Recursos e Hídricos). Meteorological stations were distant from the monitoring stations, approximately in: Missão Velha, 4.5 and 7.5 km; Brejo Santo: 18.0 and 19.0 km; Lavras de Mangabeira, 6.7 and 5.8 km; Iguatu, 15.9 km 11.7 km; Mauriti, 13.4 and 12.0 km; Jaguaribe, 18.7 km; Jaguaretama, 18 km.

## Results

**Fig. 3:**
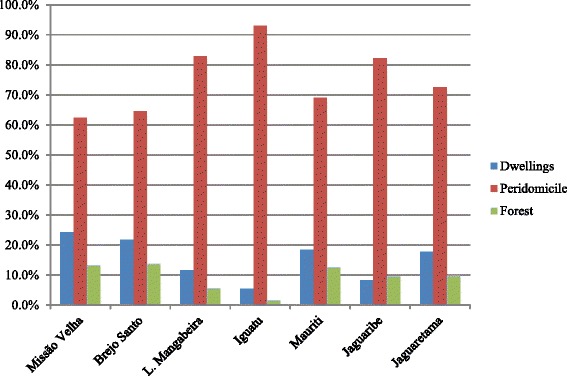
Percentage of sand fly specimens recorded in dwellings (intradomicile), peridomicile, and forest environments, considering all the seven municipalities monitored in areas under the impact of the São Francisco River Transposition Project, State of Ceará, Brazil, May 2011 to December 2014

A total of 214,213 sand fly specimens, belonging 18 species, were captured over 44 consecutive months (Table 1), with emphasis on the municipalities of Mauriti (14 species), Missão Velha (12 species), and Jaguaribe and Brejo Santo (10 species each). *Lutzomyia longipalpis* (Lutz & Neiva, 1912) specimens constituted 92.7% of the total captures, being the most abundant species in all municipalities. Over the complete set of MSs, 83.7% of the total specimens were captured in the peridomicile, 10.4% in dwellings, and only 5.9% in the forest. Considering each municipality separately, the specimens were most abundant in the peridomicile: 62.4% in Missão Velha, 64.6% in Brejo Santo, 82.9% in Lavras de Mangabeira, 93.0% in Iguatu, 69.1% in Mauriti, 82.2% in Jaguaribe and 72.5% in Jaguaretama (Fig. 3).

**Table 1:** Total number of specimens captured (N), species dominance by gender (D%) and total (Total %), species richness (S), Shannon's diversity index (H′), and monthly average for each of the seven municipalities monitored in the period between May 2011 and December 2014, in areas under the impact of the São Francisco River Water Transposition Project, state of Ceará, Brazil

Species		Missão Velha			Brejo Santo			Lavras de Mangabeira			Iguatu			Mauriti			Jaguaribe			Jaguaretama		
		*N*	D (%)	Total (%)	*N*	D (%)	Total (%)	*N*	D (%)	Total (%)	*N*	D (%)	Total (%)	*N*	D (%)	Total (%)	*N*	D (%)	Total (%)	*N*	D (%)	Total (%)
*Br. brumpti*	**♀**	0	0	0	0	0	0	0	0	0	0	0	0	0	0	0	1	0	0	0	0	0
	**♂**	0	0		0	0		0	0		0	0		0	0		1	0		0	0	
*De. samueli*	**♀**	14	0.1	0.3	1	0	0.1	0	0	0	0	0	0	0	0	0	0	0	0	0	0	0
	**♂**	30	0.2		5	0.1		0	0		0	0		1	0		0	0		0	0	
*Ev. cortelezzii*	**♀**	27	0.2	0.4	0	0	0.1	8	0	0	0	0	0	3	0	0.1	0	0	0	0	0	0
	**♂**	42	0.3		5	0.1		4	0		1	0		2	0		0	0		0	0	
*Ev. evandroi*	**♀**	331	2.1	5.4	33	0.5	1.9	390	0.9	2.6	477	0.5	1.3	83	0.9	2.5	61	0.2	0.4	10	0.1	0.2
	**♂**	541	3.4		85	1.4		737	1.7		672	0.7		151	1.6		64	0.2		21	0.1	
*Ev. lenti*	**♀**	761	4.7	14.6	131	2.1	6.6	496	1.1	3	585	0.6	1.6	238	2.6	8.2	4	0	0	4	0	0
	**♂**	1600	9.9		281	4.5		835	1.9		853	0.9		522	5.6		11	0		3	0	
*Ev. walkeri*	**♀**	15	0.1	0.1	0	0	0	0	0	0	0	0	0	0	0	0	0	0	0	0	0	0
	**♂**	8	0		0	0		0	0		0	0		1	0		0	0		0	0	
*Lu. longipalpis*	**♀**	3871	24	61.2	1522	24.2	89.1	16,529	37.6	92.5	36,065	40	95.8	2044	22.1	87	17,106	49.7	99.4	7342	52.4	99.6
	**♂**	6002	37.2		4079	64.9		24,146	54.9		50,236	55.8		5998	64.9		17,130	49.8		6609	47.2	
*Mi. oswaldoi*	**♀**	0	0	0	0	0	0	0	0	0	0	0	0	0	0	0	3	0	0	3	0	0.1
	**♂**	0	0		0	0		1	0		0	0		0	0		4	0		8	0.1	
*Mi. peresi*	**♀**	0	0	0	0	0	0	0	0	0	0	0	0	0	0	0	0	0	0	1	0	0
	**♂**	0	0		0	0		0	0		0	0		0	0		1	0		1	0	
*Mi. quinquefer*	**♀**	22	0.1	0.3	0	0	0	0	0	0	0	0	0	1	0	0	0	0	0	0	0	0
	**♂**	34	0.2		1	0		0	0		0	0		1	0		0	0		0	0	
*Mi. villelai*	**♀**	835	5.2	14.5	45	0.7	2	339	0.8	1.9	494	0.5	1.3	24	0.3	0.4	24	0.1	0.1	2	0	0
	**♂**	1501	9.3		81	1.3		498	1.1		719	0.8		14	0.2		14	0		2	0	
*Mg. migonei*	**♀**	16	0.1	0.3	6	0.1	0.2	1	0	0	0	0	0	25	0.3	1	0	0	0	0	0	0
	**♂**	40	0.2		5	0.1		3	0		0	0		68	0.7		0	0		0	0	
*Ny. intermedia*	**♀**	140	0.9	1.9	3	0	0.1	1	0	0	0	0	0	24	0.3	0.7	0	0	0	0	0	0
	**♂**	168	1		2	0		0	0		0	0		39	0.4		0	0		0	0	
*Ny. whitmani*	**♀**	35	0.2	0.5	0	0	0	0	0	0	0	0	0	3	0	0	2	0	0	0	0	0
	**♂**	42	0.3		0	0		0	0		0	0		1	0		0	0		1	0	
*Pa. abonnenci*	**♀**	0	0	0	0	0	0	0	0	0	0	0	0	0	0	0	0	0	0	0	0	0
	**♂**	0	0		0	0		0	0		0	0		1	0		0	0		0	0	
*Pa. brasiliensis*	**♀**	0	0	0	0	0	0	0	0	0	0	0	0	0	0	0	0	0	0	0	0	0
	**♂**	0	0		0	0		0	0		0	0		1	0		0	0		0	0	
*Pa. shannoni* Series	**♀**	0	0	0	0	0	0	0	0	0	0	0	0	0	0	0	1	0	0	0	0	0
	**♂**	0	0		0	0		0	0		0	0		0	0		1	0		0	0	
*Sc. sordellii*	**♀**	49	0.3	0.4	1	0	0	0	0	0	2	0	0	2	0	0	1	0	0	0	0	0
	**♂**	21	0.1		0	0		4	0		1	0		1	0		0	0		1	0	
Total		16,145			6286			43,992			90,105			9248			34,429			14,008		
S		12			10			9			6			14			10			8		
H′		0.535			0.203			0.153			0.096			0.234			0.018			0.014		
Monthly average		52.42			20.02			142.83			292.55			29.33			215.18			86.47

Considering each municipality separately, 16,145 specimens belonging to 12 species were captured in Missão Velha, of which 61.2% were *Lu. longipalpis*. This species was the most abundant in both MSs (SISA = 0.975 and SISA = 0.981). The other common species included *Evandromyia lenti* (Mangabeira, 1938) (D% = 14.6%, SISA = 0.820 and 0.737), *Micropygomyia villelai* (Mangabeira, 1942) (D% = 14.5% and SISA = 0.757 and 0.399), *Evandromyia evandroi* (Costa Lima & Antunes, 1936) (D% = 5.4% and SISA = 0.556 and 0.547), and *Nyssomyia intermedia* (Lutz & Neiva, 1912) (D% = 1.9% and SISA = 0.315 and 0.080) (Tables 1 and 2). Other species of epidemiological importance collected in this municipality with a percentage less than 1% included *Migonemyia migonei* (Franca, 1920) and *Nyssomyia whitmani* (Antunes & Coutinho, 1939).

**Table 2:** Abundance index (SISA) with final classification (FC) for each monitoring stations (MSs) of the seven municipalities monitored in the period between May 2011 and December 2014, in areas under the impact of the São Francisco River Water Transposition Project, State of Ceará, Brazil

Species	Missão Velha				Brejo Santo				Lavras de Mangabeira				Iguatu				Mauriti				Jaguaribe		Jaguaretama	
	MV1		MV2		BS1		BS2		LM1		LM2		I1		I2		M1		M2		JB		JT	
	SISA	FC	SISA	FC	SISA	FC	SISA	FC	SISA	FC	SISA	FC	SISA	FC	SISA	FC	SISA	FC	SISA	FC	SISA	FC	SISA	FC
*Br. brumpti*	–	–	–	–	–	–	–	–	–	–	–	–	–	–	–	–	–	–	–	–	0.019	vi	–	–
*De. samueli*	0.173	viii	0.013	xi	0.012	vi	0.033	vi	–	–	–	–	–	–	–	–	0.011	ix	–	–	–	–	–	–
*Ev. cortelezzii*	0.190	vii	0.088	v	0.008	viii	0.023	vii	0.009	v	0.043	v	–	–	0.006	v	0.019	viii	0.016	viii	–	–	–	–
*Ev. evandroi*	0.556	iv	0.547	iii	0.227	iv	0.293	iv	0.509	iii	0.459	iii	0.369	iii	0.372	iv	0.405	iii	0.237	iii	0.469	ii	0.219	ii
*Ev. lenti*	0.820	ii	0.737	ii	0.531	ii	0.556	ii	0.636	ii	0.650	ii	0.483	ii	0.563	ii	0.623	ii	0.656	ii	0.167	iv	0.065	iv
*Ev. walkeri*	0.057	xii	0.019	x	–	–	–	–	–	–	–	–	–	–	–	–	0.008	x	0.000	xii	–	–	–	–
*Lu. longipalpis*	0.975	i	0.981	i	1.000	i	1.000	i	1.000	i	1.000	i	1.000	i	1.000	i	0.996	i	0.997	i	0.977	i	1	i
*Mi. oswaldoi*	–	–	–	–	–	–	–	–	0.005	vii	–	–	–	–	–	–	–	–	–	–	0.062	v	0.082	iii
*Mi. peresi*	–	–	–	–	–	–	–	–	–	–	–	–	–	–	–	–	–	–	–	–	0.014	viii	0.014	viii
*Mi. quinquefer*	–	–	–	–	–	–	0.017	ix	–	–	–	–	–	–	–	–	0.021	vii	–	–	–	–	–	–
*Mi. villelai*	0.757	iii	0.399	iv	0.277	iii	0.316	iii	0.450	iv	0.398	iv	0.324	iv	0.389	iii	0.136	v	0.157	iv	0.271	iii	0.045	v
*Mg. migonei*	0.191	vi	0.047	viii	0.045	v	0.052	v	0.009	v	–	–	–	–	–	–	0.186	iv	0.149	v	–	–	–	–
*Ny. intermedia*	0.315	v	0.080	vi	0.012	vi	0.023	vii	–	–	0.009	vii	–	–	–	–	0.072	vi	0.141	vi	–	–	–	–
*Ny. whitmani*	0.098	ix	0.034	xi	–	–	–	–	–	–	–	–	–	–	–	–	–	–	0.010	xi	0.01	x	0.017	vi
*Pa. abonnenci*	–	–	–	–	–	–	–	–	–	–	–	–	–	–	–	–	–	–	0.011	ix	–	–	–	–
*Pa. brasiliensis*	–	–	–	–	–	–	–	–	–	–	–	–	–	–	–	–	–	–	0.011	ix	–	–	–	–
*Pa. shannoni* Series	–	–	–	–	–	–	–	–	–	–	–	–	–	–	–	–	–	–	–	–	0.019	vi	–	–
*Sc. sordellii*	0.077	x	0.049	vii	–	–	0.004	x	0.005	vii	0.018	vi	–	–	0.006	v	–	–	0.019	vii	0.014	viii	0.017	vi

In the Brejo Santo municipality, 6284 specimens were captured, of which 89.1% were *Lu. longipalpis*, the most abundant species in both MSs (SISA = 1.00) and all collection sites (dwellings, peridomicile and forest), followed by *Ev. lenti* (D% = 6.6% and SISA = 0.531 and 0.556), *Mi. villelai* (D% = 2.0% and SISA = 0.277 and 0.316) and *Ev. evandroi* (D% = 1.9% and SISA = 0.227 and 0.293) (Tables 1 and 2). Ten species were identified, including *Deanemyia samueli* (Deane, 1955), *Evandromyia cortelezzii* (Brethes, 1923), *Micropygomyia quinquefer* (Dyar, 1929), *Mg. migonei*, *Ny. intermedia* and *Sciopemyia sordellii* (Shannon & Del Ponte, 1927).

Nine species were found in Lavras da Mangabeira, wherein the second largest number of captured specimens occurred (*N* = 43,992); the dominant and most abundant species was *Lu. longipalpis* in both MSs (D% = 92.5% and SISA = 1.00). The other more abundant species were *Ev. lenti* (D% = 3.0% and SISA = 0.636 and 0.650), *Ev. evandroi* (D% = 2.6% and SISA = 0.509 and 0.459), and *Mi. villelai* (D% = 1.9% and SISA = 0.450 and 0.398) (Tables 1 and 2); the values are similar to those of Missão Velha and Brejo Santo.

The municipality of Iguatu exhibited both the lowest species richness (S = 6) and the highest number of captured specimens (*N* = 90,105), of which 95.8% were *Lu. longipalpis*, the most abundant species in both MSs (SISA = 1.00). The other most common species were *Ev. lenti* (D% = 1.6% and SISA = 0.483 and 0.563), *Mi. villelai* (D% = 1.3% and SISA = 0.324 and 0.389), and *Ev. evandroi* (D% = 1.3% and SISA = 0.369 and 0.372) (Tables 1 and 2).

The site with both fewer captured specimens (*N* = 9248) and greater number of species (S = 14) was Mauriti. Generally, *Lu. longipalpis* was the dominant (D% = 86.8%) and most abundant species in the two MSs (SISA = 0.996 and 0.997), and all collection sites (dwellings, peridomicile and forest), followed by *Ev. lenti* (D% = 8.2% and SISA = 0.623 and 0.656), *Ev. evandroi* (D% = 2.5% and SISA = 0.405 and 0.237) and *Mg. migonei* (D% = 1.0% and SISA = 0.186 and 0.149) (Tables 1 and 2).

Ten species and 34,429 specimens were collected in the municipality of Jaguaribe, of which 99.4% were *Lu. longipalpis*, which was also the most abundant species (SISA = 0.977). Thus, all other species collected in this municipality had percentage values lower than 1%. A similar occurrence was observed in the municipality of Jaguaretama, where 14,008 specimens belonging to eight species were captured, of which 99.6% were *Lu. longipalpis*, which was the most abundant species (SISA = 1.000) (Tables 1 and 2).

The diversity indices showed that the Missão Velha municipality had the greatest diversity (H′ = 0.535). On the other hand, Jaguaribe and Jaguaretama showed lower Shannon’s diversity index values (Table 1). These index values not only are influenced by the lower richness than that observed in other municipalities, but also reflect the strong dominance of *Lu. longipalpis*, which represented almost 100% of all specimens. However, those values did not differ significantly between MSs (*P* > 0.05) (Table 3).

**Table 3:** Student’s *t*-test (*t*) with degrees of freedom (*df*) for the Shannon’s diversity index in the seven municipalities monitored in the period between May 2011 and December 2014

	Brejo Santo		Lavras de Mangabeira		Iguatu		Mauriti		Jaguaribe		Jaguaretama	
	*df*	*t*	*df*	*t*	*df*	*t*	*df*	*t*	*df*	*t*	*df*	*t*
Missão Velha	12,080	48.145	25,577	88.831	19,655	109.66	19,411	48.015	18,978	130.333	21,673	125.974
Brejo Santo			7878	8.139	6872	18.05	13,915	4.118	6766	31.479	7294	31.523
Lavras de Mangabeira					78,103	24.136	12,403	15.085	67,332	58.949	51,413	54.037
Iguatu							10,406	26.829	107,940	47.171	33,228	40.512
Mauriti									10,194	42.318	11,214	41.995
Jaguaribe											27,997	1.979

The similarity index indicates how similar two areas are in terms of species richness. Since areas with values greater than 0.5 are considered similar, all areas in the present study were also considered, with Missão Velha and Mauriti (SI = 0.89) and Jaguaribe and Jaguaretama (SI = 0.89) the most similar to one another, whereas Brejo Santo and Jaguaribe were the least similar (SI = 0.50). The Kruskal-Wallis analysis did not indicate significant differences in the number of sand flies captured in the dwelling, peridomicile and forest from all MSs (Missão Velha: *P* = 0.100; Brejo Santo: *P* = 0.506; Lavras de Mangabeira: *P* = 0.581; Iguatu: *P* = 0.654; Mauriti: *P* = 0.451; Jaguaribe: *P* = 0.771; and Jaguaretama: *P* = 0.412).

**Fig. 4:**
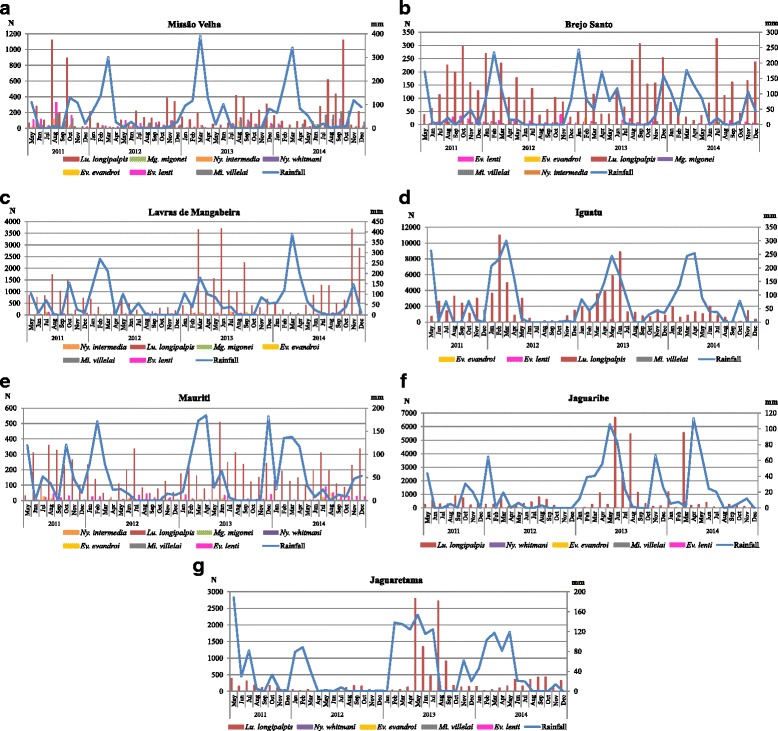
Monthly sand fly numbers (*N*) and rainfall (mm) in each of the seven municipalities: **a** Missão Velha; **b** Brejo Santo; **c** Lavras de Mangabeira; **d** Iguatu; **e** Mauriti; **f** Jaguaribe; and **g** Jaguaretama, considering most abundant species and leishmaniasis vectors, monitored in areas affected by the São Francisco River Transposition Project, State of Ceará, Brazil. Total number of specimens captured from May 2011 to December 2014

The sand fly density was correlated with rainfall, when compared with the month of capture and with previous month, and very little in the previous two months (Table 3). Sand fly fauna in the Iguatu municipality, including *Lu. longipalpis*, was influenced by regional climatic conditions: the highest population density occurred during the rainy months, with population peaks between January and June, whereas in the dry months (July to October), the population decreased. In Missão Velha, the sand fly fauna, and specifically the species *Ev*. *evandroi*, *Ev*. *lenti*, *Lu*. *longipalpis*, *Mg*. *migonei* and *Ny*. *intermedia*, showed negative correlation with rainfall. For these species, the negative correlation occurred with rainfall in the month of capture (Month 0), as well as in the previous month (Month 1), and in two previous months (Month 2). This negative correlation with rainfall also occurred with the total number of specimens (Month 1) and with *Ev. evandroi* (Month 1), *Ev. lenti* (Month 0) and *Lu. longipalpis* (Month 1) in Brejo Santo; and *Ev. evandroi* (Month 1), *Ev. lenti* (Months 0 and 1), *Mi. villelai* (Month 1) and *Mg. migonei* (Month 0) in Lavras de Mangabeira; and *Ev. lenti* (Months 0 and 1) in Mauriti. In Jaguaribe and Jaguaretama municipalities, the sand fly density, and specifically *Lu. longipalpis,* presented positive correlation with rainfall of the previous two months (Month 2) (Table 4, Fig. 4).

**Table 4:** Spearman’s correlation between the sand fly vector density (most abundant species and leishmaniasis vectors) monitored in the seven municipalities and rainfall from May 2011 to December 2014

Species	Lag Months	Spearman’s Correlation													
		Missão Velha		Brejo Santo		Lavras de Mangabeira		Iguatu		Mauriti		Jaguaribe		Jaguaretama	
		*r*	*P*	*r*	*P*	*r*	*P*	*r*	*P*	*r*	*P*	*r*	*P*	*r*	*P*
*Ev. evandroi*	0	-0.477**	0.001	-0.140	0.364	-0.256	0.093	-0.232	0.130	-0.129	0.406	0.056	0.719	-0.176	0.252
	1	-0.460**	0.002	-0.345^*^	0.022	-0.489**	0.001	-0.122	0.431	-0.226	0.141	0.109	0.482	-0.043	0.780
	2	-0.191	0.214	-0.261	0.087	-0.113	0.465	-0.348*	0.021	-0.071	0.646	0.115	0.457	0.048	0.756
*Ev. lenti*	0	-0.553**	0.000	-0.384^*^	0.010	-0.379*	0.011	-0.143	0.356	-0.141	0.361	-0.032	0.835	-0.104	0.502
	1	-0.553**	0.000	-0.112	0.471	-0.504**	0.000	-0.195	0.204	-0.521**	0.000	0.107	0.490	0.103	0.506
	2	-0.380*	0.011	-0.215	0.162	0.031	0.841	-0.155	0.314	-0.301*	0.047	0.170	0.270	0.052	0.736
*Lu. longipalpis*	0	-0.383*	0.010	-0.251	0.100	-0.046	0.765	0.413**	0.005	-0.023	0.884	-0.036	0.816	-0.011	0.943
	1	-0.581**	0.000	-0.317^*^	0.036	-0.042	0.785	0.450**	0.002	-0.030	0.846	0.143	0.354	0.161	0.295
	2	-0.465**	0.001	-0.074	0.633	0.196	0.202	0.195	0.205	-0.018	0.908	0.371*	0.013	0.391**	0.009
*Mi. villelai*	0	-0.059	0.702	-0.017	0.913	-0.205	0.181	-0.169	0.273	-0.027	0.862	0.277	0.069	-0.202	0.188
	1	-0.601**	0.000	-0.240	0.117	-0.605**	0.000	-0.227	0.138	-0.118	0.445	0.380*	0.011	0.039	0.803
	2	-0.445**	0.002	-0.149	0.334	-0.286	0.059	-0.329*	0.029	-0.072	0.643	0.397**	0.008	0.167	0.280
*Mg. migonei*	0	-0.567**	0.000	-0.246	0.107	-0.511**	0.000	–	–	-0.017	0.910	–	–	–	–
	1	-0.369*	0.014	0.005	0.976	-0.205	0.181	–	–	-0.161	0.298	–	–	–	–
	2	-0.158	0.304	0.112	0.467	-0.205	0.181	–	–	0.006	0.968	–	–	–	–
*Ny. intermedia*	0	-0.508**	0.000	0.013	0.935	-0.054	0.726	–	–	-0.183	0.234	–	–	–	–
	1	-0.376*	0.012	-0.065	0.676	0.133	0.390	–	–	-0.247	0.106	–	–	–	–
	2	-0.138	0.372	-0.044	0.779	0.199	0.195	–	–	-0.218	0.156	–	–	–	–
*Ny. whitmani*	0	-0.223	0.146	–	–	–	–	–	–	-0.205	0.181	-0.194	0.207	0.129	0.403
	1	-0.353*	0.019	–	–	–	–	–	–	-0.006	0.969	0.163	0.289	0.031	0.844
	2	-0.045	0.772	–	–	–	–	–	–	-0.115	0.458	0.248	0.104	0.263	0.084
Total	0	-0.505**	0.000	-0.262	0.086	-0.094	0.542	0.385**	0.010	-0.055	0.721	-0.026	0.866	-0.015	0.922
	1	-0.621**	0.000	-0.335*	0.026	-0.103	0.507	0.433**	0.003	-0.131	0.396	0.153	0.320	0.169	0.273
	2	-0.423**	0.004	-0.121	0.436	0.163	0.291	0.178	0.247	-0.066	0.672	0.375*	0.012	0.405**	0.006

Two-tailed test, with a 99% and 95% confidence limit

*P < 0.05, **P < 0.01

## Discussion

The environmental impacts of anthropic activities such as continuous deforestation, construction of hydroelectric plants, works and projects of different nature, migration, implementation of major agricultural projects, and military activities can provide new ACL epidemiological profiles in several American countries [1–3]. In this context, they can also contribute to the urbanization of diseases, since the transmission cycles tend to occur in dwellings and peridomicile [3].

The hypothesis that alterations caused by any environment-modifying activity might affect the population dynamics of leishmaniasis sand fly vectors should be considered. Especially, the alterations associated with deforestation and considerable environmental changes, thinking the competence of the vector in adapting to the urban environment. In this last aspect, it is known that sand flies are opportunistic and have eclectic feeding habits. In fact, all environmental change processes, in some way, are determinant in enabling a close contact between humans and leishmaniasis vectors.

The Brazil’s San Francisco River project is already a reality in Paraíba and Pernambuco, Brazilian states that are very close to Ceará. The Brazilian Government estimates that the project will be completed in Ceará in the next months. These municipalities will continue to be monitored, by the State Department of Health and by our team, to assess the effects of environmental impacts not only on *Lu. longipalpis* populations, but also on the incidence of visceral leishmaniasis in dogs and of AVL and ACL in humans.

The differences in population curves, for each municipality, could be related to some characteristics. Physiogeographic aspects and soil type can influence sand fly populations, by affecting breeding sites of immature forms.

The presence of *Lu. longipalpis* was observed in every month throughout the year, with the most visible population peaks occurring in the months following the rainy season; an exception was the Iguatu municipality, wherein the population peaks occurred during the rainy period. In this particular case, some other environmental factors may have favored this different behavior during the captures. For this species, we found correlation with rainfall in the months of capture and up to two months before. According to Deane [17], the tendency of *Lu. longipalpis* predominance in the rainy season has already been observed in the semi-arid areas of north-eastern Brazil, where populations of *L. longipalpis* increased during rainy seasons. However, in other biogeographic areas, this pattern may change, for example, in eastern Costa Rica, an endemic area of AVL, where the climate is hot and rainfall low [18], similar to the north-eastern Brazil, where greater abundance of *Lu. longipalpis* occurs in the dry season. It should also be considered that the establishment of this vector in peridomicile areas depends on some factors such as abundant organic matter for the development of larvae and availability of food sources for adults.

In fact, it is still questionable whether the density of sand fly vector species has an impact on the occurrence of human cases. Keeping in mind that high density of the vector can increase the number of specimens infected with *Leishmania* spp. and thereby increase the risk of transmission, this is a hypothesis that should not be disregarded. The records and population densities of *Lu. longipalpis*, the main AVL vector in Brazil, stand out for their relevance in all municipalities investigated. The strong dominance of this sand fly as compared to other species in all seven municipalities, mainly in dwellings and peridomicile, is notorious. Although highly anthropized, the seven municipalities exhibited average specific richness but low diversity of sand flies. The ability of *Lu. longipalpis* to feed often on domestic and synanthropic animals and its remarkable anthropophilia, favor its adaptation to changing environments, enabling the maintenance of the visceral leishmaniasis transmission cycle in rural areas and its spread to urban areas [19].

Early studies on AVL in Ceará were performed in the city of Sobral, where 100,000 inhabitants died during an outbreak in 1953; researchers were able to identify the vector, *Lu. longipalpis*, as well as domestic and wild reservoirs of *Leishmania* (*Leishmania*) *infantum* [17, 20]. Since then, AVL has been reported as endemic to the state. In the period corresponding to the present study (between 2011 and 2014) the highest incidence of AVL occurred in 2011, with 611 new cases. Among the investigated municipalities, those considered of intense transmission (Missão Velha, Brejo Santo and Mauriti) recorded 30, 26 and 39 cases of AVL, respectively.

Evidence from studies conducted in Serra de Baturité, Ceará, suggested *Ny*. *whitmani* as an ACL vector due to the finding of natural infection with *L*. (*V*.) *braziliensis*, the high density of the species in transmission sites, and its high degree of anthropophilia [21–23]. Further, in this location, another sand fly, *Mg*. *migonei*, abundant in dwellings, naturally infected with *L*. (*V*.) *braziliensis* [21–23] and anthropophilic behavior, was also suggested as an ACL vector. Both species may possibly share the transmission of the parasite. *Psychodopygus wellcomei* Fraiha, Shaw & Lainson, 1971 is also present in forested areas in this municipality, but so far, no evidence of its participation in the transmission of ACL has been found [24].

Among the municipalities where ACL cases were recorded over the study period, Missão Velha showed the highest number of cases (*n* = 23), 12 of which in 2014. Albeit in a smaller number, this locality recorded the occurrence of *Ny. intermedia* (*n* = 305), *Mg. migonei* (*n* = 53), and *Ny. whitmani* (*n* = 77).

Previous studies have reported a coincidental distribution of the disease and of *Ny. whitmani* and *Mg. migonei* in Ceará, suggesting these sand flies were acting as ACL vectors within the state [22, 25, 26]. Considering the other municipalities with records of ACL transmission, Iguatu did not record the presence of any of these vectors; thus, it would be interesting to investigate whether registered cases are actually coming from this municipality, and if so, to assess whether any of the species found in the municipality took part in the transmission. While *Ny. intermedia* and *Mg. migonei* occurred in Brejo Santo, Lavras da Mangabeira and Mauriti, *Ny. whitmani* was found solely in Mauriti.

In fact, environmental changes can alter the epidemiology of leishmaniasis, affecting mostly vectors and reservoirs, leading to a considerable increase in the number of people exposed to the risk of infection [1–3].

The São Francisco River Transposition Project, regarding the environmental impact and particularly the record of both AVL and ACL in directly affected municipalities, should monitor the vector population curve, and the possibility that the occupation of new habitats might increase the transmission risk area. Above all, health managers should evaluate the need for surveillance and prevention measures against these diseases.

According to the policies of the Brazilian Ministry of Health for surveillance and control of leishmaniasis [1, 2] and following the guidance from the World Health Organization [3], considering the diversity of sand fly vectors of ACL and AVL, an entomological surveillance measures have been recommended. It is important monitor environmental changes that may affect the behavior of sand fly vectors, especially near and within transmission risk areas.

## Conclusions

This study confirms the presence of several *Leishmania* spp. vectors in the seven municipalities affected by the São Francisco River Transposition Project, with *Lu. longipalpis* being the most abundant species all study sites. Vector populations in these municipalities should be monitored, ultimately to assess the associations between environmental changes and sand fly population dynamics and leishmaniasis transmission risk.

## Abbreviations

ACL: American cutaneous leishmaniasis
AVL: American visceral leishmaniasis
D%: Species dominance
H′: Shannon’s diversity index
ISA: Index of species abundance
MSs: Monitoring stations
S: Species richness
SI: Sørensen’s qualitative similarity index
SISA: Standardized index of species abundance
